# The Multiverse of Plant Small RNAs: How Can We Explore It?

**DOI:** 10.3390/ijms23073979

**Published:** 2022-04-02

**Authors:** Zdravka Ivanova, Georgi Minkov, Andreas Gisel, Galina Yahubyan, Ivan Minkov, Valentina Toneva, Vesselin Baev

**Affiliations:** 1 Institute of Molecular Biology and Biotechnologies, 4108 Markovo, Bulgaria; zivanova@plantgene.eu (Z.I.); george-minkov@uni-plovdiv.bg (G.M.); minkov@plantgene.eu (I.M.); toneva@plantgene.eu (V.T.); 2 Department of Plant Physiology and Molecular Biology, University of Plovdiv, 4000 Plovdiv, Bulgaria; gyahubyan@uni-plovdiv.bg; 3 Institute of Biomedical Technologies (ITB), CNR, 70126 Bari, Italy; andreas.gisel@ba.itb.cnr.it; 4 Center of Plant System Biology and Biotechnology, 4000 Plovdiv, Bulgaria

**Keywords:** plant small RNAs, siRNAs, microRNAs, vsiRNAs, phasiRNAs, natsiRNAs, tasiRNAs, bioinformatics tools, NGS, sRNA-seq, software, data analysis

## Abstract

Plant small RNAs (sRNAs) are a heterogeneous group of noncoding RNAs with a length of 20–24 nucleotides that are widely studied due to their importance as major regulators in various biological processes. sRNAs are divided into two main classes—microRNAs (miRNAs) and small interfering RNAs (siRNAs)—which differ in their biogenesis and functional pathways. Their identification and enrichment with new structural variants would not be possible without the use of various high-throughput sequencing (NGS) techniques, allowing for the detection of the total population of sRNAs in plants. Classifying sRNAs and predicting their functional role based on such high-performance datasets is a nontrivial bioinformatics task, as plants can generate millions of sRNAs from a variety of biosynthetic pathways. Over the years, many computing tools have been developed to meet this challenge. Here, we review more than 35 tools developed specifically for plant sRNAs over the past few years and explore some of their basic algorithms for performing tasks related to predicting, identifying, categorizing, and quantifying individual sRNAs in plant samples, as well as visualizing the results of these analyzes. We believe that this review will be practical for biologists who want to analyze their plant sRNA datasets but are overwhelmed by the number of tools available, thus answering the basic question of how to choose the right one for a particular study.

## 1. Structural Diversity—The Big Bang of Small RNAs in Plants

Since the discovery that double-stranded RNAs (dsRNAs) can trigger gene silencing in *Caenorhabditis elegans* [1], small RNAs (sRNAs) have become recognized in plants as key molecules for maintaining plant genome integrity and regulating plant development and stress responses. Plant sRNAs are short molecules of various lengths (typically 20–24 nucleotide—nt), processed from single-stranded hairpin RNA or double-stranded RNA precursors [2,3,4]. Next-generation sequencing (NGS) techniques have greatly expanded the possibilities for identifying sRNAs by providing a massive number of sequences from a single plant sample. Additionally bioinformatics and computational tools play a vital role in these growing technologies, being an essential part of identifying and quantifying sRNAs and understanding the world of plant sRNAs. Advances in this field have led to a better understanding of the different types of sRNA as well as the various complex roles and interactions these molecules have within the context of gene expression.

**Figure 1:**
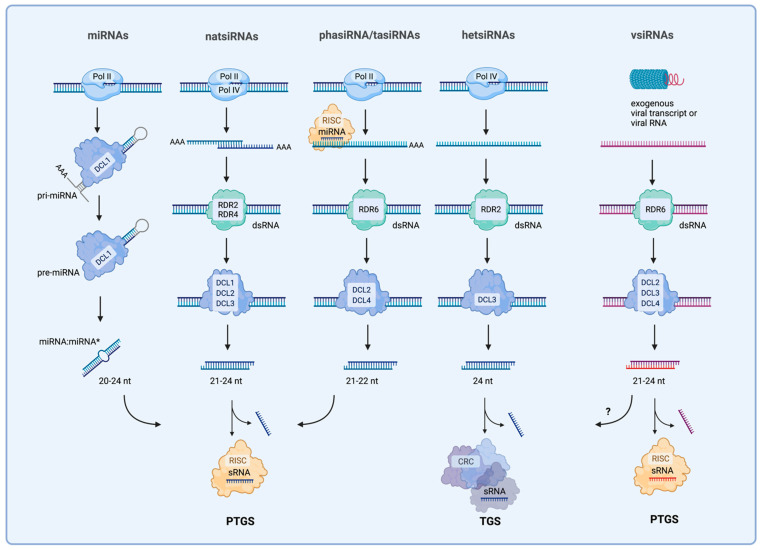
Biogenesis pathways of endogenous and exogenous small RNAs in plants. A majority of miRNAs are encoded by their own genes and transcribed by Pol II. Primary transcripts (pri-miRNA) are processed by DCL1, producing the miRNA hairpin precursor (pre-miRNA), which is further cleaved by DCL1 to generate a miRNA:miRNA* duplex. Unlike miRNAs, the other plant small RNAs are not encoded by genes. Instead, natsiRNAs originate from overlapping transcripts; secondary small RNAs originate from noncoding or coding loci, or TE; and hetsiRNA are transcribed from TE and repeat elements of pericentromeric chromatin. Downstream, the resulting transcripts (including the exogenous viral transcripts and viral RNAs) fall into processing pathways to generate double-stranded RNAs by RDRs, which are cleaved into small RNAs by DCLs. Small RNAs are loaded into an RNA-induced silencing complex (RISC), triggering posttranscriptional gene silencing—PTGS (miRNAs, natsiRNAs, secondary siRNA, vsiRNAs)—or into chromatin remodelling complexes (CRC) to induce transcriptional gene silencing—TGS—by RNA-dependent DNA methylation—RdDM (hetsiRNAs, vsiRNAs). (Created with BioRender.com, accessed on 27 March 2022).

Plant endogenous sRNAs are classified into two major types: microRNAs (miRNAs) and small-interfering RNAs (siRNAs). The latter group includes several different subtypes: natural antisense siRNAs (natsiRNAs), secondary siRNAs (phasiRNAs and tasiRNAs), and heterochromatic siRNAs (hcsiRNAs). Exogenous sRNAs, virus- or viroid-genome-derived siRNAs (vsiRNAs), on the other hand, are products of a host plant’s RNA silencing pathway and have been implicated in the plant’s defense response (Figure 1) [5,6,7].

Many miRNA-encoding genes (MIR) have been found in the intronic regions of so-called “host genes” within plant genomes, as well as in other genomic loci. They are transcribed by RNA polymerase II, forming a long primary RNA transcript (pri-miRNA) [8]. Part of the pri-miRNA sequence folds into a perfect stem–loop structure, stabilized by the RNA-binding protein DDL, to form a precursor miRNA (pre-miRNA). The precursor is recognized by an endoribonuclease Dicer-like (DCL1), cleaved to the miRNA:miRNA* duplex, and exported to the cytoplasm [9,10,11]. Finally, the mature (canonical) miRNA is loaded into the RNA-induced gene silencing complex (RISC), which guides an ARGONAUTE 1 protein (AGO1) to target mRNAs. Recent studies have shown that miRNA* may not necessarily be degraded as previously thought, but rather have a functional role—to recognize specific target genes. It has been found that most miRNA target genes of miRNAs are transcription factors (TF) [12,13].

As a result of inaccurate Dicer processing, pre-miRNAs can produce miRNAs:miRNA* duplexes shifted from the canonical miRNA, resulting in the generation of so-called isomiRs. This phenomenon is not only observed as a result of an enzymatic error, but isomiRs are molecules that seem to be programmed to be generated. It is also evidenced by their presence in various tissues and under specific environmental conditions, where their expression levels may differ accordingly [14]. Due to the altered production, and therefore depending on their sequence, the targets of isomiR molecules may or may not differ from mature miRNA targets [15].

Unlike miRNAs, which have relatively defined genes in the plant genome, siRNAs can be generated from ideally double-stranded RNAs (dsRNAs), generated from a variety of sources, such as the following: RNAs transcribed from inverted repeats; natural cis-antisense transcript pairs; genome-rich loci with retroelements; or even from an exogenous viral source. Depending on the specific catalytic activity of DCLs, these dsRNAs may be cleaved into molecules of different lengths, usually 21–24 nt. For example, natsiRNAs originate from dsRNAs derived either from overlapping transcripts (cis-natsiRNAs) or from highly complementary transcripts derived from different loci (trans-natsiRNAs) that form dsRNAs and are cleaved by DCL1, DCL2, or DCL3 [16,17,18].

The biogenesis of secondary siRNAs—phased siRNAs (phasiRNAs), including trans-acting siRNAs (tasiRNAs)—is more complex, involving Pol II transcription of noncoding or protein-coding loci as well as transposable elements. Producing the secondary, RNA-dependent RNA polymerase, RDR-dependent sRNAs, requires transcript targeting by miRNAs [19]. Cleaved targets are converted into dsRNA by RDR6 and cut by DCL2 and DCL4 to siRNAs of size 21 or 22 nt. We should point out that the production of secondary siRNAs (phasiRNAs and tasiRNAs) from mRNAs, noncoding RNAs, requires miRNAs to target mRNA sources. The name of trans-acting RNAs (tasiRNAs), found in some types of phasiRNAs, comes from their ability to function as miRNAs in a homologously dependent manner, directing the cleavage of mRNAs other than those at their source.

Both exogenous derived and endogenous sRNAs can guide transcriptional gene silencing (TGS) or posttranscriptional gene silencing (PTGS). miRNAs can regulate many biological processes by gene silencing at the PTGS level [20]. They pair with target mRNAs initiating cleavage or inhibition protein translation [20]. miRNAs, natsiRNAs, and phasiRNAs (including tasiRNAs) function primarily at the PTGS level via cleavage or translational suppression of target transcripts; although, several findings have been reported indicating that they can induce DNA methylation [21,22].

Like miRNAs, siRNAs are loaded into AGO-containing RISC complexes in order to guide target silencing at PTGS. The 24 nt hcsiRNAs (sometimes known as heterochromatic sRNA (hetsiRNAs) or repeat-associated sRNAs (rasiRNAs)) are the most abundant sRNAs in plants, responsible for inducing transcriptional silencing of transposons and pericentromeric repeats via RdDM. The generation of hctsiRNAs requires Pol IV transcription, followed by dsRNA synthesis by RDR2 and processing by DCL3 (Figure 1). Usually, hcsiRNAs promote DNA or histone modifications at the loci which produce them, including retrotransposons, 5S rDNA, and centromeric repeats (the reason some authors call them rasiRNAs) [23,24,25,26].

vsiRNAs are produced when exogenous viral transcripts are converted to dsRNAs by various mechanisms, including RDRs, with the help of DCL4, DCL2, and DCL3. These vsiRNAs are loaded into RISC complexes and eventually slice the hostile viral transcripts or the target host mRNAs [27,28,29].

## 2. Functional Diversity—The Expanse of the sRNAs World

Functional analysis of miRNAs showed their crucial involvement in many plant biological processes by regulating various genes at PTGS. These small but powerful molecules have been widely studied and found to play a key role in various development processes, including, among others, the following: meristem boundary identity [30,31,32]; auxin signaling [33,34]; organ separation, leaf development [35,36,37]; lateral root formation [38,39,40]; juvenile-to-adult vegetative phase [41,42,43]; leave development [35,36,37]; floral organ development [33,34,35,36]; flowering time [36,43,44]; control of cell growth and proliferation [45,46].

The functional role of miRNA also includes plant responses to biotic and abiotic stresses. Environmental changes can trigger plants to adapt their miRNA expression or produce new miRNAs to cope with stress. Various stress-dependent miRNAs have been identified and annotated in plants under numerous abiotic stress conditions, including: hypoxia and oxidative stress [47,48,49,50]; drought [51,52,53,54]; nutrient homeostasis [50,55,56,57,58,59,60,61,62]; cold [53,63,64,65]; heat [19]; salinity [53,66]; UV-B radiation [57,67]; mechanical stress [68]; heavy metals [69]. Furthermore, some miRNAs are linked to biotic responses in bacterial and fungal interactions with plants. For example, plants effectively utilize miRNAs to finetune their phytohormone pathways, along with genes involved in pathogenic virulence [70].

The discovery of isomiRs has highlighted the functional significance of miRNAs in gene regulation. Their altered sequences, as compared to canonical mature miRNA and miRNA*, may lead to a new set of target molecules, adding another level of complexity to miRNAs function [68,71]. In addition, some, if not all, miRNAs may reversibly interact with their targets, suggesting that miRNAs may regulate their own biogenesis pathways and supporting the possibility that miRNA regulatory roles may not be limited to protein-encoding transcripts. [72].

Although some sRNAs may still be unknown or less studied, other classes have important functional roles in plants. Secondary sRNAs (phasiRNAs and their subgroups of tasiRNAs) are known or predicted to function in various biological processes. However, given the many PHAS loci and phasiRNA members found in different plants, their exact functional role remains poorly documented overall. Triggered by miRNAs, these secondary sRNAs are found to be involved in several important biological processes. tasiRNAs can target the pentatricopeptide repeat-containing genes (PPR), many of which are abiotic-stress-related genes. Recent findings suggest that some tasiRNAs are involved in the thermotolerance of plants through the regulation of heat-stress-related TF [73]. Other tasiRNAs can target ARFs, which are related to the auxin-mediated control of developmental processes, including leaf morphogenesis, developmental timing, lateral root growth, and somatic embryogenesis [74,75,76,77,78,79,80,81]. Another relatively highly conserved tasiRNA, TAS4, is related to MYBs regulation [82], which is associated with lignin biosynthesis, bioflavonoid biosynthesis, and fruit development [82,83]. Different studies suggested that phasiRNAs are associated with plant immunity [84,85,86], as they can target disease resistance genes [87,88,89]. Interestingly phasiRNAs are also involved in plant parasitism. The authors in [90] found that a parasitic plant uses trans-species silencing to repress transcripts within the host plant, thereby facilitating its parasitism.

Some cis-natural antisense transcripts (cis-NATs) have been reported to generate natsiRNAs in response to abiotic and biotic stresses [16,91,92,93] or to accumulate in specific developmental stages [17,94]. natsiRNAs are the least studied small RNAs in terms of their functional roles in plants; however, some studies suggest that their role may be related to various mechanisms of plant development and stress response, such as pathogen resistance [95], salt tolerance [5], and cell wall biosynthesis [96].

Although most sRNAs function through PTGS, some perform their biological role through TGS through the RdDM mechanism. The hcsiRNAs are very important in maintaining genome stability and gene regulation as they induce epigenetic modification in repeat elements. Plant genomes consist of large portions of such repeats [97], having the ability of genome jumping and multiplication, causing gene disruption. Such events require plants to have protective mechanisms, where hcsiRNAs step in and prevent mobilization of the transposons. Repeats are also located in the promoter regions of protein-coding genes, which generate 24 nt sRNAs. hcsiRNAs can control these regulatory elements via RdDM mechanism, the methylation status of which can also affect the downstream gene expression [98]. Another functional role of hcsiRNAs is their participation in plant reproduction, including methylation programs of the gamete cells observed within the endosperm and zygote [99].

The interactions between host plants, viruses, and various abundant vsiRNAs are highly complex. vsiRNAs, produced through the host RNA silencing pathways, have been implicated in the host defense response [100]. Studies point out that the disease symptoms in the infected plant are a consequences of RNA silencing directed against important host genes by the same vsiRNAs [101,102]. They can induce antiviral defense through PTGS or TGS of viral RNA, as well as hijack the host’s RNA silencing system, in order to target complementary host transcripts.

## 3. Bioinformatics Tools for Exploration and Analysis of the World of Small RNAs

In the post-genomics era, NGS technologies provide quantitative evaluation and single-base resolution of known and novel sRNAs through various sRNA-seq methods. Various computational tools have been developed to analyze sRNA NGS data, allowing for not only the detection, profiling, and annotation of different classes of sRNAs, but also comparing the sRNA expression levels between samples. Over the past decade, these bioinformatics methods and software applications have become an inseparable part of exploring the sRNAs world, creating a need for more user-friendly tools, viable for biologists without programming knowledge or advanced bioinformatics skills. Most of the tools that analyze sRNA-seq data are run on Linux/Unix servers or clusters. This often requires knowledge of command-line workflows and interpreting large amounts of data files, which can often be a difficult task for biologists. A user-friendly graphic interface is a must for such software.

The next pitfall is that some tools require complex installations, dependencies, and third-party modules requiring further IT knowledge, which may disrupt the smooth analysis process. Furthermore, some tools are also tailored to operate on a specific operating system, which adds another layer of complexity. While some tools attempt to address these disadvantages by providing direct web access with a GUI and no upfront setup requirement, this has its own flaws. Users may need to work with large data files, which can be hard to upload and store, or use-sensitive, proprietary data, which cannot be stored off site. In conclusion, almost every bioinformatics tool has its pros and cons, and therefore it is crucial to select the right tool for each application. We provide a review of recently developed tools for sRNAs data analysis as a support for people searching help to enter in the world of sRNA or keep updated in this fast-developing field.

In this current review, we have gathered more than 35 tools developed over the past five years that deal with the analysis of sRNAs, including miRNAs, isomiRs, natsiRNA, phasiRNAs, and vsiRNAs, with most using sRNA-seq data as their primary source of sRNAs from the wet-lab experiment (Table 1). Not surprisingly, a significant part of these tools are dedicated to miRNAs as being the most famous and explored sRNA class. These miRNA-related tools have a variety of purposes—profiling and annotation of miRNAs and their isomiRs; identification of new MIR genes and putative precursors; discovery of miRNA targets and miRNA–mRNA interactions.

There is no unified protocol for analyzing miRNAs and their isoforms from sRNA-sequencing data. Nevertheless, most tools use similar major processing stages. Usually, the discovery stage includes several preprocessing steps of the raw reads from the sRNA-seq data, including sequencing library QC, adapter trimming, and cleaning low-quality reads, size filtering, etc. These steps generally integrate various widely accepted third-party tools that are integrated into the main analysis software, for example, FastQC, Prinseq, Cutadapt, FASTX and Trimommatic, TrimGalore, etc. (IsomiR_Window, miRDis, miRPursuit, MiRkwood, MirGalaxy, IsoMiRmap, miRge, sRNAnalyzer, isomiR2Function, MirCure, QuickMIRSeq). Next, the clean reads are mapped to a reference database in order to recognize known miRNAs or their isoforms across the processed samples. Here, the majority of the tools used Bowtie for third-party aligner, though some used others such as PatMaN (miRCat2, miRPursuit) and BWA (miRKwood, sRNAnalyser). The most frequently used reference miRNAs databases used are miRBase and miRGene [140,141,142].

It is worth noting that some tools can process unique molecular identifiers (UMIs) if the source files require that analysis protocol (sRNAbench, miRge, sRNAtools). When it comes to normalizing and quantifying the miRNAs identified in samples by differential expression analysis, most of the developed software uses external specially dedicated statistical packages, such as DESeq2, EdgeR, and EBSeq (SRNAbench, MirGalaxy, IsoMiRmap, miRge, IsomiR_Window, isomiR2Function, SRNAbench, miRDis, isomiR2Function, etc.), or RPM normalization (QuickMIRSeq, SRIS, sRNAnalyzer, PmiRDiscValie). Recently, the isomiR phenomenon has attracted the attention of researchers. Due to their potential importance, an increasing number of tools have provided distinctive features, such as comprehensive isomiR exploration, including further specific profile analysis (sRNAbench, miRGalaxy, isoMiRmap, miRge, IsomiR_Window, sRNAnalyser, isomiR2Function, miRDis, QuickMIRSeq, etc.).

Besides discovering known miRNAs, the identification of novel plant MIR genes is also an essential part of the comprehensive sRNAs analysis. Recently, various computational tools have been developed for identifying miRNAs with the help of supporting data from next-generation sequencing datasets, along with applying criteria based on the features of the miRNA biogenesis. Usually, these tools use aligners, such as Bowtie or ParMaN, to map reads to reference sequences or genome (miRKwood, miRDeep-P2, miRge, miRCat2, microRPM) and then searches for clusters of sRNAs that can be produced from potential precursor as miRNA:miRNA*. These clusters are carefully examined on various criteria to ensure they are consistent with miRNA biogenesis rules. Putative precursors are passed to a third-party package for thermodynamic calculation and RNA folding, for which most tools use ViennaRNA [143]. Some tools employ the help of the machine learning algorithms to strengthen their discovery approaches (miRDetect, miRHunter, miRge, microRPM, iwa-miRNA). The standard approach to discover new miRNA genes and their precursor is using sRNA-seq data and a reference genome. Still, some specific tools can use different NGS data (PmiRDiscVali) or even perform analysis without a reference genome (Mirnovo, miRDetect, microRPM), which can include other than sRNA-seq data, or support sRNA analysis of non-model plants where only a low-quality or even no reference genome exists.

Recently, different bioinformatics tools, such as Targetfinder [144], psRNATarget [120], comTAR [145], psRobot [146], CleaveLand [147,148], and sPARTA [149], have been developed to predict miRNA targets in plants, and were reviewed before. These tools have since been implemented in other, newer miRNA software, where they serve a similar purpose. There are not many new tools for target discovery, especially for plants. The psRNATarget tool was updated with the ability to use NGS data, along with an improved scoring schema for target site prediction, including a better-weighted method for the mismatch-sensitive “seed” region. This tool also considers mRNA target accessibility, i.e., the energy required to open mRNA secondary structure near the target, by calculating unpaired energy (UPE), using the most popular tools of the ViennaRNA package [150]. TarHunter is a new development that relies on orthologous miRNA clustering from desired species and cross-species conservation filters and implementing RNAhybrid (ViennaRNA) to search for miRNA targets. Importantly, target discovery may take advantages of novel approaches, including not only sRNA-seq data but also degradome sequences (SRIS, TarHunter, PAREsnip2). Degradome sequences represent fragments of mRNA cleaved by miRNAs or siRNA [151,152,153,154,155]. Usually, to discover potential target sites, degradome sequences are mapped to a transcriptome using Bowtie in order to provide information for the 5’ cleavage of the mRNA [156].

miRNA-dedicated tools seem to rule the computational universe when it comes to sRNAs exploration. Nevertheless, there are a number of software packages dedicated to identifying other sRNAs (natsiRNAs, phasi- and tasi-RNAs, tRNA-derived sRNAs, 24 nt siRNA, etc.). Their profiling often includes the same computational stages mentioned above, such as preprocessing of sRNA data and mapping, etc. Here, the miRNA reference databases are extended beside miRbase, with Ensembl, RfamBD, RefSeq, tRNA, and rRNA databases, and genomes with already specific annotations for small RNAs (unitas, SRIS, sRNAtools, sRNAbench, SRNAnalyser). In addition to broad profiling of sRNAs, there are some more specific tools for exploring phasiRNAs annotation (PHASIS, PhasiRNAanalyser, unitas), which are based on sequence homology instead of particular biogenesis rules.

The PHASIS suite provides comprehensive tools for de novo prediction and characterization of PHAS loci, emphasizing plants, where these loci are numerous. The recently developed PhasiRNAnalyzer also can provide identification of all crucial components in phasiRNAs’ regulatory pathway, along with furthermore verification of the interactions between phasiRNAs and their target genes based on degradome data. Moreover, the tool can perform differential expression analysis of phasiRNAs on each PHAS gene locus between different samples. natsiRNAs can be predicted with the tool NATpare. It requires sRNA, transcriptome, and optionally degradome data as input and enables the identification of both cis- and trans-natsiRNAs. The tool identifies potential NAT pairs and potential natsiRNAs, and, if degradome data is provided, the candidate natsiRNAs are subject to functional analysis using PAREsnip2 [125] to search for potential mRNA targets.

Virus infections are recognized as a significant threat to agricultural production and plant health. Efficient and accurate detection of vsiRNAs and therefore of viruses and viroids in plants is essential for the development of effective strategies to manage the spread and impact of viral diseases. For this reason, some bioinformatics tools with the ability to detect these exogenous small RNAs (VirusDetect, sRNAprofiler, SRIS, VSD toolkit) have recently been developed. This detection is carried out by mapping sRNA-seq data to viroid and/or virus sequence repositories by Bowtie, BWA, or BLAST. Additionally, some of the tools provide the ability to assemble and reconstruct the viral genome by gathering all vsiRNAs to aid in the discovery of new pathogen species or strains (using third-party assemblers such as SPades, Velvet, etc.).

## 4. Conclusions and Future Perspectives

Plant research is increasing slowly keeping up with popular research target, such as animals or humans, visible also in how many bioinformatics tools and algorithms became available in recent years for analysing sRNAs in plants. Due to the relatively complex biology of sRNAs, these tools are far from perfect and request further development to approach questions about the creation, involvement, and regulation of sRNA. Most of the tools have their pros and cons and can tackle a specific argument for a specific type of sRNA.

Currently, most of the tools are designed to handle miRNA analysis, leaving a gap for tools dedicated to other plant sRNAs species. This is because the research for miRNA is much more advanced than other sRNAs and can help to train and validate new algorithms. Another bottleneck is that many of these tools still require significant bioinformatics skills, creating difficulties in applicability and usability. Unfortunately, we are still confronted with the fact that, rather than unifying standards of analysis input and outputs, some software applications diverge further by implementing their own bespoke formats, which cannot be exchanged between tools. Nevertheless, computational tools that can bring comprehensive solutions and support wet-lab processes are needed now more than ever to expand our knowledge of sRNAs in revealing the full scope of their universe in a user-friendly way for basic research, but also for application in agronomy and breeding.

## Figures and Tables

**Table 1:** List of bioinformatics tools for plant small RNA analysis and their main features.

miRNA and isomiR Tools:	
**Tool Name**	1. IsomiR_Window
**Type**	Local, VM
**Description**	**Features**: isomiRs identification; identification of noncoding RNAs; miRNA prediction; miRNA and isomiR quantification and functional analysis. **Third-party tools**: Bowtie, DEseq2, MirDeep2, miRDP2, Miranda, TargetFinder, etc. **Output**: visualizations and results as tables and interactive graphs.
**Ref. and tool URL**	Vasconcelos et al. [103] https://github.com/andreiaamaral/IsomiR-Window/, accessed on 27 March 2022
**Tool Name**	2. PAREameters
**Type**	Local
**Description**	**Features**: miRNA-mRNA interaction analysis; small RNA and degradome data; non-model organisms. **Third-party tools**: miRCat2, miRPlant, PAREsnip2. **Output**: set of data-inferred thresholds for a rule-based prediction of miRNA–mRNA interactions.
**Ref. and tool URL**	Thody et al. [104] https://github.com/sRNAworkbenchuea/UEA_sRNA_Workbench, accessed on 27 March 2022
**Tool Name**	3. QuickMIRSeq
**Type**	Local, Linux
**Description**	**Features**: miRNA and isomiR quantification. **Third-party tools and DB**: miRBase **Output**: User friendly report; Rich visualisation via set of QC metrics and plots.
**Ref. and tool URL**	Zhao et al. [105] https://sourceforge.net/projects/quickmirseq/files/, accessed on 27 March 2022
**Tool Name**	4. Iwa-miRNA
**Type**	Web, Galaxy
**Description**	**Features**: miRNA annotation; miRNA quantification. **Third-party tools and DB**: miRDeepP2 and miRCat2; miRBase, PmiREN, sRNAanno, and PsRNA. **Output**: genomic distribution, expression of miRNAs and their host genes, subgenome bias, genomic duplication and miRNA expansion, SNP effect.
**Ref. and tool URL**	Zhang et al. [106] http://iwa-mirna.omicstudio.cloud/, accessed on 27 March 2022
**Tool Name**	5. miRCat2
**Type**	Local, MAC, Linux and Windows
**Description**	**Features**: miRNA prediction. **Third-party tools**: RNAFold (Vienna RNA Package), PatMaN. **Output**: graphical representation of the hairpin structures and plots of sequence alignments of secondary structure.
**Ref. and tool URL**	Paicu et al. [107] https://github.com/sRNAworkbenchuea/UEA_sRNA_Workbench, accessed on 27 March 2022
**Tool Name**	6. Mirnovo
**Type**	Web, Local, Mac, Linux
**Description**	**Features**: miRNA prediction; miRNA precursor prediction; non-reference genome support. **Third-party tools**: Bowtie2, Random Forest R package. **Output**: distribution of all feature values (coverage, sequence complexity and genomic) and visualization by QC-plots.
**Ref. and tool URL**	Vitsios et al. [108] https://github.com/dvitsios/mirnovo, accessed on 27 March 2022
**Tool Name**	7. MiRPursuit
**Type**	Local, Linux, Unix, Mac
**Description**	**Features**: miRNA identification; tasiRNAs identification; non-model organisms. **Third-party tools**: FASTQC, FASTX; UEA sRNA Workbench; PatMaN; UEAsRNA Workbench (miRProf, miRCat); UEA sRNA Workbench (tasiPredictor). **Output**: detailed report from the outputs of each process in miRPursuit along with a matrix with the raw counts exportable to other programs.
**Ref. and tool URL**	Chaves et al. [109] https://github.com/forestbiotech-lab/miRPursuit, accessed on 27 March 2022
**Tool Name**	8. isomiR2Function
**Type**	Local, Linux, Mac
**Description**	**Features**: isomiR identification; templated and non-templated isomiRs; isomiR quantification; target identification. **Third-party tools**: DSeq and Ebseq. **Output**: detailed isomer identification report; provides support for the visualisation of read mapping on corresponding precursor sequences.
**Ref. and tool URL**	Yang et al. [110] https://github.com/347033139/isomiR2Function, accessed on 27 March 2022
**Tool Name**	9. TarHunter
**Type**	Local, Linux
**Description**	**Features**: miRNA target identification; degradome data support. **Third-party tools**: UBLAST, MUSCLE, USEARCH. **Output**: files, containing all predicted miRNA targets.
**Ref. and tool URL**	Ma et al. [111] https://github.com/XMaBio, accessed on 27 March 2022
**Tool Name**	10. MirCure
**Type**	Local, Linux, MAC OS
**Description**	**Features**: miRNA prediction; miRNA annotation. **Third-party tools**: DNApi, Cutadapt, Bowtie2, Samtools, Genomic Alignments R package. **Output**: calculated score of secondary structures, gene expression, graphical visualizations.
**Ref. and tool URL**	Ylla et al. [112] https://github.com/ConesaLab/MirCure, accessed on 27 March 2022
**Tool Name**	11. PlantMiRP-Rice
**Type**	Local, Linux, Win
**Description**	**Features**: miRNA prediction; rice pre-miRNA prediction. **Third-party tools**: miRBase, PlantGDB databases, RNAFold. **Output**: three-column contents of the identifier, predicted label (positive or negative), and corresponding score for each testing sample.
**Ref. and tool URL**	Zhang et al. [113] https://github.com/yygen89/riceMirP, accessed on 27 March 2022
**Tool Name**	12. mirKwood
**Type**	Web, Galaxy, Docker, Local, Unix
**Description**	**Features**: miRNA identification. **Third-party tools**: Bowtie2 or BWA; miRbase; RNAFold. **Output**: web page where miRNA precursors are displayed in a table; various formats: CSV, FASTA, GFF, text report in ORG mode and read clouds.
**Ref. and tool URL**	Guigon et al. [114] https://bioinfo.cristal.univ-lille.fr/mirkwood/mirkwood.php, accessed on 27 March 2022
**Tool Name**	13. miRLocator
**Type**	Local, Win, MacOS, Linux, Docker, web
**Description**	**Features**: miRNA prediction. **Third-party tools**: Vienna RNA package; MiRBase; miRNEST. **Output**: predicted miRNA and its corresponding passenger strand for the tested pre-miRNA sequence.
**Ref. and tool URL**	Zhang et al. [115] https://github.com/cma2015/miRLocator, accessed on 27 March 2022
**Tool Name**	14. StarSeeker
**Type**	Phyton, Local
**Description**	**Features**: miRNA identification. **Third-party tools and DB**: miRbase. **Output**: putative miRNA sequence given the precursor and the mature sequences.
**Ref. and tool URL**	Natsidis et al. [116] https://biopython.org/, accessed on 27 March 2022
**Tool Name**	15. miRHunter
**Type**	Web
**Description**	**Features**: miRNA precursor identification; comparative and non-comparative *ab initio* prediction. **Third-party tools**: BLAST, RNAFold. **Output**: potential pre-miRNAs
**Ref. and tool URL**	Koh et al. [117] https://repository.hanyang.ac.kr/handle/20.500.11754/114034, accessed on 27 March 2022
**Tool Name**	16. sRNAnalyzer
**Type**	Local, Linux
**Description**	**Features**: miRNA identification; miRNA SNPs detection. **Third-party tools**: Bowtie, Fastx_toolkit, Prinseq, Cutadapt, MirGeneDB. **Output**: detailed feature and profile text files.
**Ref. and tool URL**	Wu et al. [118] http://srnanalyzer.systemsbiology.net/, accessed on 27 March 2022
**Tool Name**	17. mirGalaxy
**Type**	Web, Docker, Mac, Win, Linux
**Description**	**Features**: miRNA identification; templated and non-templated isomiRs identification; miRNA and isomiR quantification **Third-party tools**: Galaxy, TrimGalore, FastQC, Bowtie, DeSeq2, EdgeR. **Output**: detailed reports from the differential expression analysis, PDF report, charts and plots visualization.
**Ref. and tool URL**	Glogovitis et al. [119] https://hub.docker.com/r/glogobyte/mirgalaxy, accessed on 27 March 2022
**Tool Name**	18. psRNATarget
**Type**	Web
**Description**	**Features**: miRNA target identification. **Third-party tools**: RNAup program in Vienna package, BioGrid platform. **Output**: comprehensive list of small RNA/target pairs with ranking scores.
**Ref. and tool URL**	Dai et al. [120] http://plantgrn.noble.org/psRNATarget/, accessed on 27 March 2022
**Tool Name**	19. PlantMirP2
**Type**	Local, Docker, Web
**Description**	**Features**: miRNA prediction; miRNA precursor prediction. **Third-party tools and DB**: miRbase. **Output**: charts and plots visualizations.
**Ref. and tool URL**	Fan et al. [121] https://github.com/wuqiansibai/plantMiRP2/releases/tag/v1.0/, accessed on 27 March 2022
**Tool Name**	20. PmiRDiscVali
**Type**	Local, Perl
**Description**	**Features**: miRNA prediction, degradome data support. **Third-party tools**: miRDeep-P, SVG, bowtie, ViennaRNA package. **Output**: predicted miRNA precursors and a visual representation of their secondary structure.
**Ref. and tool URL**	Yu et al. [122] https://github.com/unincrna/pmirdv, accessed on 27 March 2022
**Tool Name**	21. miRDeep-P2 (update)
**Type**	Local, Linux
**Description**	**Features**: miRNA identification. **Third-party tools**: Bowtie, ViennaRNA package. **Output**: putative pre-miRNA with folded structure, sRNA mapping to pre-miRNA visualization.
**Ref. and tool URL**	Wang et al. [123] https://sourceforge.net/projects/mirdp2/, accessed on 27 March 2022
**Tool Name**	22. PAREsnip2
**Type**	Local
**Description**	**Features**: sRNA target identification, degradome data support. **Output**: in CSV format, transcript peak information, visual representation of the sRNA–mRNA duplex.
**Ref. and tool URL**	Thody et al. [124] https://github.com/sRNAworkbenchuea/UEA_sRNA_Workbench/, accessed on 27 March 2022
**Tool Name**	23. miRDis
**Type**	Web
**Description**	**Features**: miRNA identification; miRNA prediction; miRNA quantification. **Third-party tools**: BLAST, RNAfold, infernal, EdgeR, FASTQC, Cutadapt. **Output**: summary, candidate list, annotation details, differential analysis, heatmaps.
**Ref. and tool URL**	Zhang et al. [125] http://sbbi-panda.unl.edu/miRDis/download.php, accessed on 27 March 2022
**Tool Name**	24. miRDetect
**Type**	Local, Python
**Description**	**Features**: miRNA prediction; miRNA precursor prediction; plant EST dataset support. **Third-party tools**: BLAST standalone, ViennaRNA package, **Output**: list of identified putative miRNA precursors.
**Ref. and tool URL**	Ayachit et al. [126] https://github.com/Garima268/miRDetect, accessed on 27 March 2022
**Tool Name**	25. microRPM
**Type**	Local, Perl
**Description**	**Features**: miRNA prediction; non-model organisms; non-reference genome support. **Third-party tools**: Bowtie; Vienna RNA; Trinity; LibSVM; Structure RNA sequences. **Output**: miRNA mature duplex report.
**Ref. and tool URL**	K. C. Tseng et al. [127] http://microrpm.itps.ncku.edu.tw/, accessed on 27 March 2022
**Tool Name**	26. miRge3.0
**Type**	Local, Docker, Python
**Description**	**Features**: miRNA identification; isomiR identification; miRNA and isomiR quantification; miRNa prediction; UMIs support. **Third-party tools**: Cutadapt, Bowtie, ViennaRNA, SAMtools, biopython, sklearn, numPy, SciPy, reportlab, DESeq2. **Output**: summary report, files with count reads for each miRNA species, isomiR results in GFF3, BAM for visualization.
**Ref. and tool URL**	Patil et al. [128] https://github.com/mhalushka/miRge3.0, accessed on 27 March 2022
**natsiRNA tools**	
**Tool Name**	1. NATpare
**Type**	Java, Mac, Win, Linux
**Description**	**Features**: natsiRNAs prediction and identification; degradome data support. **Third-party tools**: PARESnip2, UEA sRNA Workbench. **Output**: candidat natsiRNA, comma-separated value (CSV) format.
**Ref. and tool URL**	Thody et al. [129] https://github.com/sRNAworkbenchuea/UEA_sRNA_Workbench/, accessed on 27 March 2022
**phasi/tasiRNA tools**	
**Tool Name**	1. PhasiRNAnalyzer
**Type**	Web
**Description**	**Features**: phasiRNAs prediction; phasiRNAs target genes prediction. **Output**: list of predicted PHAS genes, phasiRNA clusters and phase-initiators.
**Ref. and tool URL**	Fei et al. [130] https://cbi.njau.edu.cn/PPSA/, accessed on 27 March 2022
**vsiRNA tools**	
**Tool Name**	1. sRNAProfiler
**Type**	Local, MacOS, Unix, Windows
**Description**	**Features**: vsiRNA identification; viroid sRNA mapping. **Output**: summary of the sRNA mapping data and graphical visualization.
**Ref. and tool URL**	Adkar-Purushothama et al. [131] https://github.com/paviudes/vbind, accessed on 27 March 2022
**Tool Name**	2. VirusDetect
**Type**	Local, Linux
**Description**	**Features**: vsiRNA identification; virus sRNA mapping; virus assembly. **Third-party tools**: BWA, Velvet, BLASTN, BLASTX. **Output**: sequences of detected viruses in fasta format.
**Ref. and tool URL**	Zheng et al. [132] http://virusdetect.feilab.net/cgi-bin/virusdetect/vd_download.cgi, accessed on 27 March 2022
**Tool Name**	3. VSD toolkit
**Type**	Web
**Description**	**Features**: vsiRNA identification; vsiRNA assembly; hcsiRNAs assembly; virus and viroid detection. **Third-party tools**: FASTx, fastqc, ConDeTri, SPAdes, CAP3, BLAST, Bowtie. **Output**: detected viruses and viroids, vsiRNAs covarage across viral genomes, assembly of virus and viroids.
**Ref. and tool URL**	Barrero et al. [133] https://github.com/muccg/yabi, accessed on 27 March 2022
**Misc. tools**	
**Tool Name**	1. sRNAtools
**Type**	Web, Docker
**Description**	**Features**: miRNA identification; isomiR identification; piRNA identification; natsiRNA identification; other sncRNAs identification and annotation; sncRNA function analysis; sncRNA quantification. **Third-party tools**: Cutadapt, FASTX-Toolkit, Bowtie, miRD-eep, Mireap, Tapirhybrid, Targetfinder, RNAhybrid, miRanda. **Output**: sncRNA list, statistics and graphics, sncRNA differential expression, targets genes.
**Ref. and tool URL**	Liu et al. [134] https://bioinformatics.caf.ac.cn/sRNAtools, accessed on 27 March 2022
**Tool Name**	2. sRIS (Small RNA Illustration System)
**Type**	Web, Linux
**Description**	**Features**: miRNA identification; vsiRNA identification; sRNA characterization; target identification; degradome data support. **Third-party tools and DB**: Bowtie, FASTX toolkit, Rfam, miRbase, microRPM. **Output**: sRNA library statistics, miRNA and vsiRNA profiles, sRNA-target profiles, genomic hotspots of vsiRNAs, miRNA heatmap, etc.
**Ref. and tool URL**	Tseng et al. [135] http://sris.itps.ncku.edu.tw/, accessed on 27 March 2022
**Tool Name**	3. Unitas
**Type**	Local, Linux, Mac, Windows
**Description**	**Features**: miRNA annotation, phasiRNA identification, piRNA annotation. **Third-party tools and DB**: Ensembl, miRBase, GtRNAdb, SILVA rRNA, piRNA cluster databases. **Output**: sequence length distribution, miRNA annotation table, sRNA annotation summary, phasiRNA annotation, etc.
**Ref. and tool URL**	Gebert et al. [136] https://sourceforge.net/projects/unitas/, accessed on 27 March 2022
**Tool Name**	4. SPORTS1.0
**Type**	Local, Linux
**Description**	**Features**: tsRNAs, rsRNAs, piRNA analysis. **Third-party tools**: Cutadapt; MiRDeep2; miRBase, rRNA database, GtRNAdb, piRNA, Ensembl, Rfam databases. **Output**: sRNA annotation, sRNA length distribution, sRNA summary, mismatch summary, tRNA, piRNA, ncRNA visualizations.
**Ref. and tool URL**	Shi et al. [137] https://github.com/junchaoshi/sports1.1, accessed on 27 March 2022
**Tool Name**	5. SCRAM
**Type**	Local, Docker
**Description**	**Features**: sRNA alignment and visualization **Output**: plots and visualizations
**Ref. and tool URL**	Fletcher et al. [138] https://sfletc.github.io/scram/, accessed on 27 March 2022
**Tool Name**	6. sRNAbench and sRNAtoolbox (update)
**Type**	Web server, Docker
**Description**	**Features**: miRNA identification; isomiR identification; miRNA quantification; sncRNA; vsiRNA, etc. **Third-party tools and DB**: miRbase, miRGeneDB, Deseq2, edgeR and UpsetR. **Output**: interactive heatmaps, box-plots, volcano-plots, genome mapping visualization, consensus tables and graphical representation of differential expression.
**Ref. and tool URL**	Aparicio-Puerta et al. [139] https://arn.ugr.es/srnatoolbox/, accessed on 27 March 2022

## Data Availability

Not applicable.
